# Identification of chromosomes in *Triticum aestivum* possessing genes that confer tolerance to the synthetic auxin herbicide halauxifen-methyl

**DOI:** 10.1038/s41598-020-65434-x

**Published:** 2020-05-26

**Authors:** Olivia A. Obenland, Dean E. Riechers

**Affiliations:** Department of Crop Sciences, University of Illinois, Urbana, IL USA

**Keywords:** Polyploidy in plants, Plant molecular biology, Agricultural genetics, Abiotic

## Abstract

Natural tolerance in hexaploid bread wheat (*Triticum aestivum* L.) to synthetic auxin herbicides is primarily due to rapid metabolic detoxification, but genes encoding these herbicide-detoxifying enzymes have yet to be identified. Herbicide safeners are commonly applied in wheat to achieve herbicide tolerance by inducing the expression and activity of herbicide-detoxifying enzymes. While safeners have been utilized for decades, knowledge of mechanisms that induce gene expression is limited. Our objective was to identify wheat chromosomes possessing genes that endow natural or safener-induced tolerance to halauxifen-methyl (HM), a postemergence (POST) wheat-selective synthetic auxin herbicide, using alien substitution (the S genome of *Aegilops searsii*) and aneuploid lines. Two POST rates of HM were applied to seedlings with 1-2 leaves (Zadoks stages 11-12), and the highest HM rate was also applied with the safener cloquintocet-mexyl (CM). Wheat chromosomes possessing genes associated only with natural HM tolerance were identified because *Ae. searsii* is HM-sensitive but CM-responsive. Lines with substitutions for 5A and 5B displayed sensitivity to HM, and experiments with nullisomic-tetrasomic (NT) lines further indicated major genes associated with HM tolerance are present on 5A and 5B chromosomes. However, the genes on 5A appear to play a larger role because lines lacking 5A chromosomes displayed more sensitivity than lines lacking 5B. Overall, these results can be utilized to guide future transcriptome analyses to identify candidate genes that confer HM tolerance in wheat.

## Introduction

*Triticum aestivum* L. (wheat) is an allohexaploid (2n = 6x = 42; AABBDD) consisting of three homoeologous sets of seven chromosomes in the A, B, and D genomes^1,2^. Since wheat is a polyploid, it can tolerate aneuploidy (the loss or duplication of chromosomes) without losing fertility, which led to the creation of aneuploid lines from the cultivar, ‘Chinese Spring’, by Dr. Ernie Sears^3–5^. Additionally, wheat alien substitution lines were created since wheat can tolerate the replacement of native chromosomes with homoeologous chromosomes from another species (referred to as the ‘alien’ genome)^6^. Current applications of aneuploid and alien substitution lines include identifying chromosomes that possess genes of interest as well as analyzing homoeologous gene expression patterns and regulation^7^. These genetic resources have allowed researchers to study a myriad of genes related to grain nutrient content, biotic and abiotic stresses, and defense compound and phytohormone biosynthesis^8–16^. Generally, these experiments are designed to identify which aneuploid and/or alien substitution line(s) lack a phenotypic response to a treatment or gene expression pattern that is normally observed in wheat. Lines with replacements or deletions of entire or portions of certain chromosomes that lack the wild-type phenotypes or expression patterns provide evidence for the location of genes of interest. Occasionally genes from the alien chromosomes enhance certain traits, particularly disease resistance, and thus allow researchers to identify genes in related species that can be transferred into hexaploid wheat for crop improvement^14,16^.

**Figure 1:**
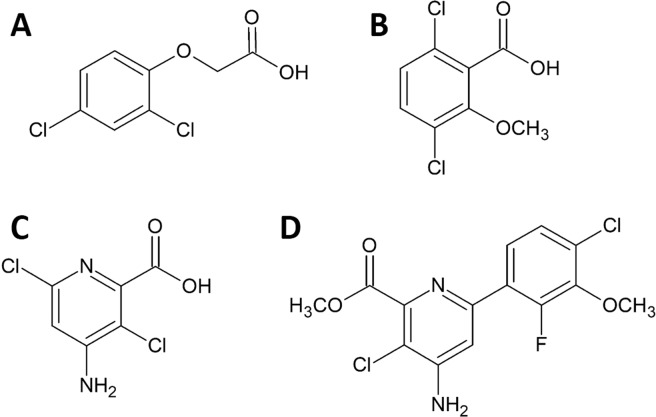
Structures of common synthetic auxin herbicides: 2,4-D (phenoxy-acetic acid; **A**), dicamba (benzoic acid; **B**), aminopyralid (pyridine carboxylic acid; **C**), and halauxifen-methyl (6-aryl-picolinic acid; **D**).

Synthetic auxin herbicides (Fig. 1) are commonly used for postemergence (POST) dicot weed control in wheat and other cereal crops^17^. These herbicides are denoted ‘synthetic auxins’ because they mimic the natural phytohormone, indole-3-acetic acid (IAA), which regulates almost every aspect of plant growth and development^18,19^. Like other cereal crops, wheat possesses a natural tolerance to synthetic auxin herbicides, which allows for selective control of dicot weeds^20^. Metabolism of herbicides to non-phytotoxic compounds is the primary mechanism for selectivity between monocots and dicots^20^. Monocots possess cytochrome P450-dependent monooxygenases (P450s) that catalyze irreversible ring-hydroxylation reactions of synthetic auxin herbicides that lead to detoxification^21^. While ring hydroxylation of synthetic auxin herbicides (such as 2,4-D) occurs in dicots to some extent^22^, metabolism of these herbicides in dicots occurs primarily through reversible reactions, such as amino acid or sugar conjugation of the carboxylic acid, which do not lead to permanent detoxification^17,21^.

P450s are detoxification enzymes that generally hydroxylate or de-alkylate the parent herbicide^23,24^, forming a less toxic compound and predisposing the herbicide to glucose conjugation by UDP-dependent glucosyltransferase (UGTs) and subsequent sequestration to the vacuole by ATP-binding cassette transport proteins^25^. While the role of certain P450s in herbicide metabolism in tolerant crops and resistant weeds has been established^24,26^, a specific gene encoding a P450 responsible for synthetic auxin metabolism has not been characterized in wheat. However, the maize (*Zea mays*) *Nsf1* and rice (*Oryza sativa*) *CYP81A6* genes encode P450s that confer tolerance to ALS-inhibiting herbicides and bentazon; *Nsf1* also confers tolerance to dicamba as well as other POST herbicides from a total of five site-of-action groups^27–29^. These findings indicate that herbicide tolerance in wheat might also be due to a single pleiotropic gene encoding a P450 enzyme that metabolizes multiple herbicides.

A common practice to increase herbicide tolerance in large-seeded cereals is to utilize herbicide safeners, which are chemical compounds that induce the expression of herbicide detoxification and transporter enzymes^30–32^. Several safeners are currently available for cereal crops to protect against herbicides that inhibit acetolactate synthase, acetyl-CoA carboxylase, and very-long-chain fatty acid elongases^33–35^. Wheat safeners are often applied POST in a tank mixture with the herbicide, which offers simplicity to applicators^32^. While safeners have been prevalent in agriculture for decades and their phenotypic and metabolic effects are well documented, knowledge of safener regulation of corresponding genes or signaling pathways is still limited^33,36^.

The herbicide examined in the present study is halauxifen-methyl (HM), which is a recently commercialized synthetic auxin and the first member of the 6-aryl-picolinic acid subclass^37,38^. HM is typically applied POST to wheat in a tank mixture that includes other herbicides and a safener^39^. Currently, the literature pertaining to HM metabolism in plants is scarce aside from de-esterification of HM to the active halauxifen-acid, followed by *O*-demethylation on the aryl ring and glucose conjugation^40^. In general, however, little is known about the genes conferring synthetic auxin tolerance and the signaling pathways needed to achieve the safener phenotype in wheat. Using a combination of wheat aneuploid and alien substitution lines, our research objectives were to (1) identify wheat chromosomes containing genes endowing natural or safener-induced tolerance to HM, and (2) identify which homoeologs identified in Objective 1 possess the relevant genes of interest. Only a few studies have been conducted using wheat aneuploid and alien substitution lines to identify chromosomes containing genes encoding herbicide-metabolizing enzymes^41,42^, but these studies did not identify genes associated with POST synthetic auxin herbicide tolerance.

## Materials and Methods

### Chemicals and plant materials

Chemicals used in the following experiments include the wheat safener, cloquintocet-mexyl (CM; formulated as a 25% active ingredient wettable powder), and the Elevore formulation of halauxifen-methyl (HM). Seed for ‘Chinese Spring’, 21 alien substitution lines and nullisomic-tetrasomic (NT) lines were acquired from the Kansas State Wheat Genetics Resource Center, and *Aegilops searsii* (PI 599163) seed was acquired from the National Small Grains Collection of the US Department of Agriculture/Agricultural Research Service. *Ae. searsii* (2n = 2x = 14; SS) is a wild diploid species and wheat relative native to the sub-Mediterranean regions of Israel, Jordan, Syria, and Lebanon^43,44^.

### Seed sowing and treatment application

Prior to germination, seeds were subjected to a cold treatment by placing them on water-soaked filter paper in Petri dishes in a 5 °C cold room for three days in order to promote uniform germination. When seeds germinated they were planted in 382 cm^3^ pots containing a 1:1:1 soil mixture of soil, peat, and sand, and slow-release fertilizer (Everris Osmocote Classic 13-13-13; BFG Supply, Burton, OH). The pots were moved to a greenhouse room with a 14 hour day length and a constant 21 to 23 °C temperature band. When seedlings produced 1-2 leaves (Zadoks stages 11-12), treatments were applied using a compressed air research sprayer calibrated to deliver 187 L ha^−1^ at 275 kPa with an even flat-fan nozzle.

### Screening alien substitution lines and nullisomic-tetrasomic (NT) lines

The first study included ‘Chinese Spring’, 21 alien substitution lines, and *Ae. searsii* in order to determine which chromosomes contain genes necessary for natural or safener-induced HM tolerance. Rates for HM were based on a typical field rate of the commercial product Quelex, which is approximately 5 g a.e. ha^−1^ ^45^. Treatments included a control (only adjuvants), 20 g a.e. ha^−1^ HM (4x), 60 g a.e. ha^−1^ HM (12x), and 60 g a.e. ha^−1^ HM with 3.75 g ai ha^−1^ (60 μM; equivalent to the field rate in Quelex) of CM (12x + CM). All treatments also included 1.25% (v/v) methylated seed oil and 2.5% (v/v) ammonium sulfate. Rates of 20 and 60 g a.e. ha^−1^ are approximately 4 and 12 times the amount of the HM field rate, respectively. Four plants from each line were subjected to each treatment, and following the application all plants were arranged in a completely randomized design in the greenhouse room described above. At 21 days after treatment (DAT) plants were cut at the soil line, bagged and dried in a 65 °C oven. After plants were dry, weights were recorded and treatments among each of the lines were compared to their respective controls. This experiment was conducted twice and data were pooled for further statistical analysis as described below. Mean biomass from the 4x, 12x, and 12 + CM treatments are expressed as a percent of the control treatment for each line.

Results for the first experiment prompted the next experiment that included ‘Chinese Spring’ and the NT lines: N5A-T5B, N5A-T5D, N5B-T5D, N5D-T5A, and N5D-T5B. Seed for the N5B-T5A line was not available from the Kansas State Wheat Genetics Resource Center. Experimental methods for this study were identical to the previous study with a few modifications: 20 g a.e. ha^−1^ HM treatment was not applied and five plants from each line were subjected to each treatment.

### Statistical analysis

For each experiment, data were pooled from each individual replication and residuals were tested for normality and homogeneity of variance with PROC UNIVARIATE and PROC GLM, respectively (SAS 9.4). Data were subjected to ANOVA and treatment means were separated using Fisher’s Protected LSD test at α = 0.01.

## Results and Discussion

### Response of alien substitution lines to halauxifen-methyl in the greenhouse

Significant injury was only noted in ‘Chinese Spring’ following the 12x treatment when compared to the nontreated control, but injury was prevented with the 12x + CM treatment (Table 1; Supplementary Fig. S1). In *Ae. searsii*, significant injury was achieved with both the 4x and 12x treatments when compared to the nontreated control, and the 12x + CM treatment was significantly different from 12x and control treatments (Table 1; Supplementary Fig. S1). These results indicate that HM injury in ‘Chinese Spring’ can be prevented when CM is included but only partially prevented in *Ae. searsii* (Table 1). The 4x and 12x HM treatments caused approximately 19% and 29% reductions in biomass in ‘Chinese Spring’, respectively, while the same treatments caused 83% and 85% reductions in biomass in *Ae. searsii*, respectively (Table 1). Overall, ‘Chinese Spring’ displayed an expected natural tolerance to HM, even at rates higher than recommended by the product label. By contrast, results for *Ae. searsii* indicate it lacks natural HM tolerance, meaning that substitutions from this species should not increase tolerance in ‘Chinese Spring’ and allow for detection of wheat chromosomes possessing genes that govern natural HM tolerance. However, these results also indicate that *Ae. searsii* possesses CM-responsive genes whose expression enhances HM metabolism, resulting in a significant reduction in injury compared to HM alone. Since it is unknown which chromosomes in *Ae. searsii* possess genes required for the safener response, this experimental approach would not likely indicate which chromosomes possess genes responsible for safener-induced HM tolerance. This is evident since all the alien substitution lines responded to the CM treatment with increased HM tolerance (Table 1). Using aneuploid lines, such as ditelosomic or nullisomic lines, or a wheat alien substitution line with foreign chromosomes from a CM-unresponsive species would be needed to identify the chromosomes and genes necessary for the safening phenotype in wheat.

**Table 1:** Mean biomass of wheat ‘Chinese Spring’, *Ae. searsii*, and 21 alien substitution lines following halauxifen-methyl and halauxifen-methyl plus cloquintocet-mexyl treatments.

Line	Nontreated Control (g)		Percent of Nontreated Control					
			4x		12x		12x + CM	
**Mean Biomass**								
‘Chinese Spring’	1.55	a	81%	ab	71%	b	90%	a
*Ae. searsii*	0.46	a	17%	b	15%	b	48%	c
1A	1.18	a	102%	a	88%	a	103%	a
1B	1.50	a	81%	ab	69%	b	74%	ab
1D	1.19	a	95%	a	82%	a	95%	a
2A	1.02	a	93%	a	79%	a	85%	a
2B	1.21	a	90%	a	83%	a	97%	a
2D	1.42	a	89%	a	83%	a	92%	a
3A	0.96	a	80%	b	80%	b	83%	ab
3B	0.64	a	95%	a	81%	a	86%	a
3D	0.75	a	88%	a	87%	a	93%	a
4A	1.16	a	90%	a	85%	a	94%	a
4B	1.20	a	96%	a	73%	b	87%	ab
4D	1.20	a	92%	ab	76%	b	96%	ab
5A	1.12	a	63%	b	59%	b	80%	a
5B	1.35	a	84%	a	53%	b	81%	a
5D	1.34	a	85%	ab	74%	b	93%	a
6A	1.41	a	82%	ab	79%	b	85%	ab
6B	1.24	a	98%	a	86%	a	81%	a
6D	1.26	a	81%	ab	75%	b	89%	ab
7A	1.31	a	86%	ab	82%	b	89%	ab
7B	1.25	a	82%	a	82%	a	91%	a
7D	1.26	a	94%	a	87%	a	90%	a

Treatments applied to seedlings (Zadoks stages 11–12) included the following: nontreated control (adjuvants included in all other treatments: 1.25% methylated seed oil and 2.5% ammonium sulfate); 20 g a.e. ha^−1^ halauxifen-methyl (HM) (4x); 60 g a.e. ha^−1^ HM (12x); and 60 g a.e. ha^-1^ HM with 3.75 g ai ha^-1^ (equivalent to 60 µM; Quelex field rate) of cloquintocet-mexyl (CM) (12x + CM). Means for the nontreated control treatment are expressed in grams (g) and means for the 4x, 12x, and 12x + CM treatments are expressed as a percentage of the nontreated control for each line. Means for each line that share the same letter indicate that the means are not significantly different (α = 0.01).

The majority of the alien substitution lines displayed similar biomass reductions as ‘Chinese Spring’ in response to the 4x (approximately 20% or less) and 12x HM (approximately 30% or less) treatments, and in general the 12x + CM treatments were not significantly different from the control (Table 1). While several lines displayed significant biomass reductions in response to either 4x or 12x HM treatments, only substitutions for the group 5 chromosomes displayed biomass reductions of approximately 40% or more for one or both HM treatments (Table 1). Substitutions for 5A and 5B displayed the largest biomass reductions among the 21 substitution lines from the 12x treatment but only the 5A line displayed a significant biomass reduction and injury from the 4x treatment (Table 1; Supplementary Fig. S2), indicating that genes on chromosome 5A primarily govern HM tolerance in hexaploid bread wheat. By contrast, biomass reductions and injury in the 5D line were more comparable to CS and other alien substitution lines (Table 1; Supplementary Fig. S2), suggesting that only chromosomes 5A and 5B possess major genes controlling HM tolerance.

### Response of nullisomic-tetrasomic lines to halauxifen-methyl in the greenhouse

**Figure 2:**
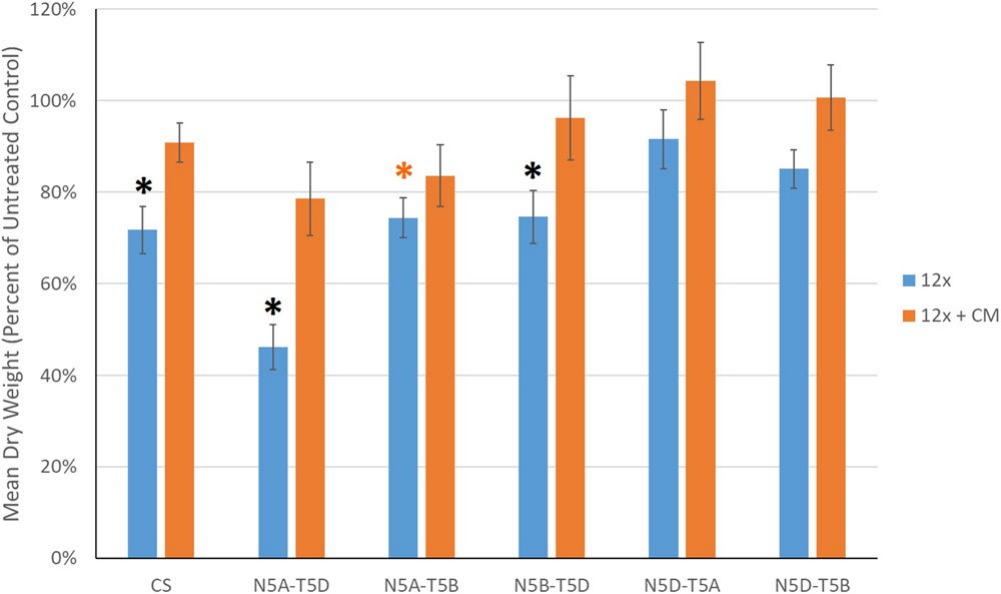
Means for nullisomic-tetrasomic (NT) lines treated with 60 g a.e. ha^−1^ halauxifen-methyl (HM) (12x; blue bars) and 60 g a.e. ha^−1^ HM with 3.75 g ai ha^−1^ (equivalent to 60 µM; Quelex field rate) of cloquintocet-mexyl (CM) (12x + CM; orange bars). Treatment means are expressed as a percent of the nontreated control for each line. A black asterisk indicates treatment means were significantly different from the nontreated control and the 12x + CM treatment. An orange asterisk indicates treatment means were significantly different from the nontreated control but not the 12x + CM treatment. All 12x + CM treatment means were not significantly different from respective nontreated control means. The means of each treatment were separated with Fisher’s LSD (α = 0.01).

Similar to the previous study, ‘Chinese Spring’ displayed significant injury (i.e., an approximate 28% biomass reduction) from the 12x treatment but injury was prevented with CM (Fig. 2). For each line, the 12x + CM treatment was not significantly different from the nontreated control, indicating that genes required for the safening phenotype are not located on group 5 chromosomes or that group 5 homoeologous genes can functionally compensate for one another. The N5A-T5D line displayed the greatest sensitivity to the 12x treatment (approximate 54% biomass reduction) relative to the nontreated control, indicating the importance of genes on chromosome 5A for natural HM tolerance (Fig. 2). Biomass reductions for the N5A-T5B and N5B-T5D line from the 12x treatment were similar to ‘Chinese Spring’ with approximate 26% and 25% biomass reductions, respectively (Fig. 2). Biomass reductions for N5D-T5A and N5D-T5B for the 12x treatment were less than ‘Chinese Spring’ with approximately 8% and 15% biomass reductions, respectively (Fig. 2). Overall, these findings are consistent with results using the alien substitution lines (Table 1) and indicate that only chromosomes 5A and 5B possess major genes that contribute to natural HM tolerance. However, we hypothesize that the genes on 5A are more important for controlling HM tolerance in wheat due to the greater level of injury resulting from the 12x treatment in N5A-T5D compared to N5B-T5D, but it is evident that 5B moderately contributes to HM tolerance since the N5A-T5B line displayed less sensitivity than N5A-T5D from the 12x treatment (Fig. 2), possibly due to functional redundancy and compensation between the 5A and 5B homoeologous chromosomes.

## Conclusions

Reductions in HM tolerance were quantified in wheat when chromosomes 5A and 5B were not present in either alien substitution lines with the S genome or in NT lines, indicating these chromosomes contain genes necessary for HM tolerance. To determine whether rates of HM metabolism are altered in these NT or alien substitution lines, metabolism studies using excised leaves can be conducted using HPLC or LC-MS methods to directly test our hypothesis. Furthermore, the information and plant materials in this study can be used to identify candidate genes responsible for HM tolerance in wheat by mining RNA-seq data generated from HM- or CM-treated leaf tissue^46^. Potential candidate genes might encode P450s or UGTs, which are typically responsible for synthetic auxin herbicide metabolism in cereals^21,23^. In addition, candidate P450 genes on wheat group 5 chromosomes may represent homologs of *Nsf1* (located on maize chromosome 5) and *CYP81* *A6* (located on rice chromosome 3), which encode P450 enzymes that confer tolerance to multiple herbicides, including dicamba in the case of the *Nsf1*^27–29^. The discovery of synteny between chromosome 3 in rice and wheat group 5 chromosomes^47–49^ supports our working hypothesis. Alternatively, it remains possible that HM tolerance in wheat is a quantitative trait rather than a qualitative trait, where major and minor genes contribute to whole-plant HM tolerance. Finally, if subsequent research determines that HM-metabolizing enzymes encoded by these genes are capable of metabolizing additional herbicides or other xenobiotics (i.e., mycotoxins, allelochemicals, or environmental pollutants), then these genes could serve as useful markers to assist breeding programs aimed at increasing abiotic or biotic stress tolerance in wheat.

## Supplementary information

Supplementary information.
