# Supplementary material for: Identification of chromosomes in Triticum aestivum possessing genes that confer tolerance to the synthetic auxin herbicide halauxifen-methyl
Source: Sci Rep. 2020 May 26;10:8713. doi: 10.1038/s41598-020-65434-x (PMC7250930; doi:10.1038/s41598-020-65434-x)
Supplement: Supplementary file 1 — Supplementary information. [file 41598_2020_65434_MOESM1_ESM.pdf]

Supplementary information

Identification of chromosomes in *Triticum aestivum* possessing genes that confer tolerance to the synthetic auxin herbicide halauxifen-methyl.

Olivia A. Obenland & Dean E. Riechers*

Department of Crop Sciences, University of Illinois, Urbana, Illinois 61801, USA.
Correspondence and requests for materials should be addressed to D.E.R. (E-mail: riechers@illinois.edu)

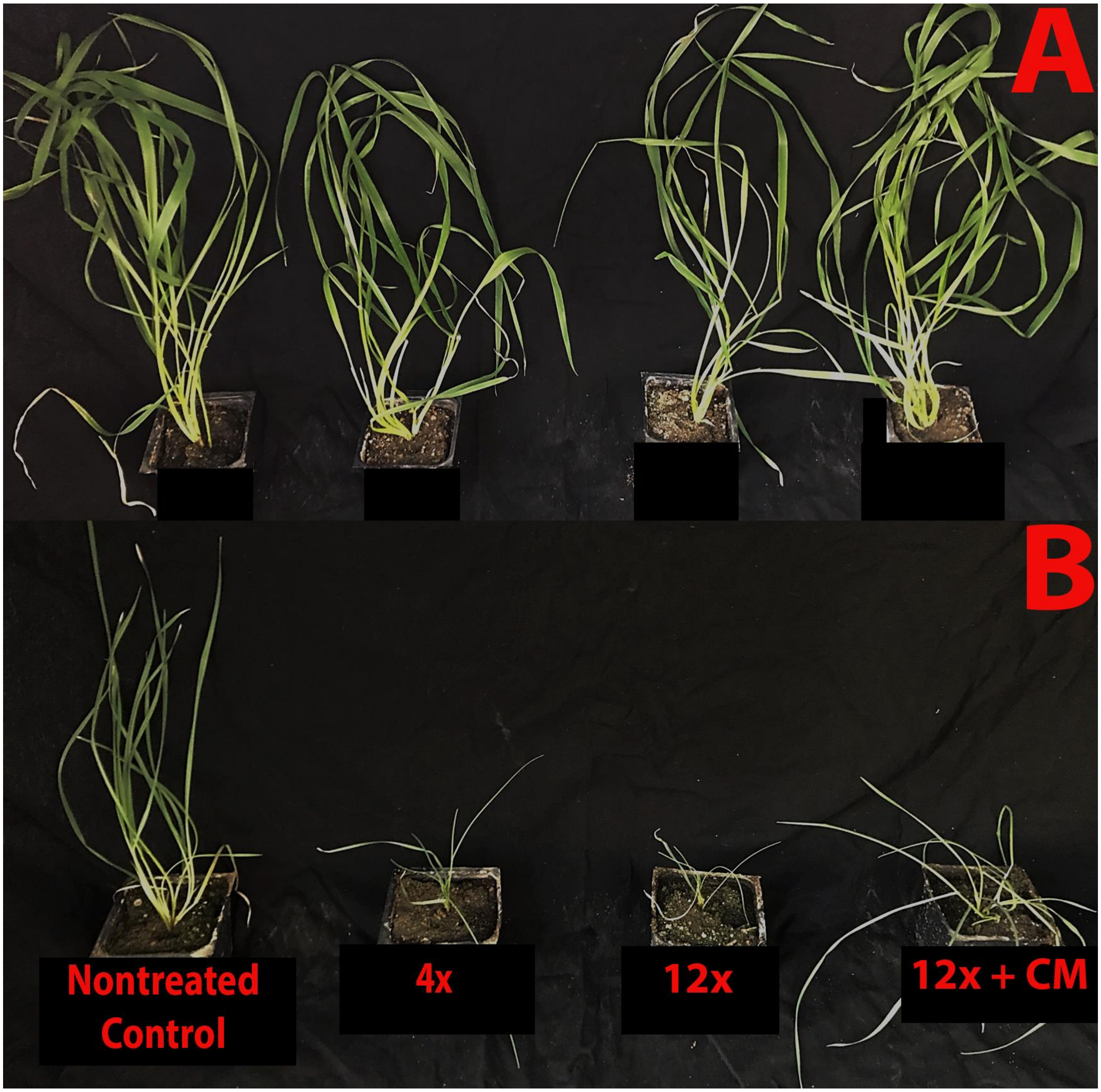

Supplementary Figure S1. Wheat ‘Chinese Spring’ (**A**) and *Ae. searsii* (**B**) plants 20 days following herbicide or herbicide plus safener treatments. Treatments were applied to seedlings at Zadoks stages 11-12 and included the nontreated control (adjuvants included in all other treatments: 1.25% methylated seed oil and 2.5% ammonium sulfate); 20 g a.e. ha^{-1} halauxifen-methyl (HM) (4x); 60 g a.e. ha^{-1} HM (12x); and 60 g a.e. ha^{-1} HM with 3.75 g ai ha^{-1} (equivalent to 60 µM; Quelex field rate) of cloquintocet-mexyl (CM) (12x + CM).

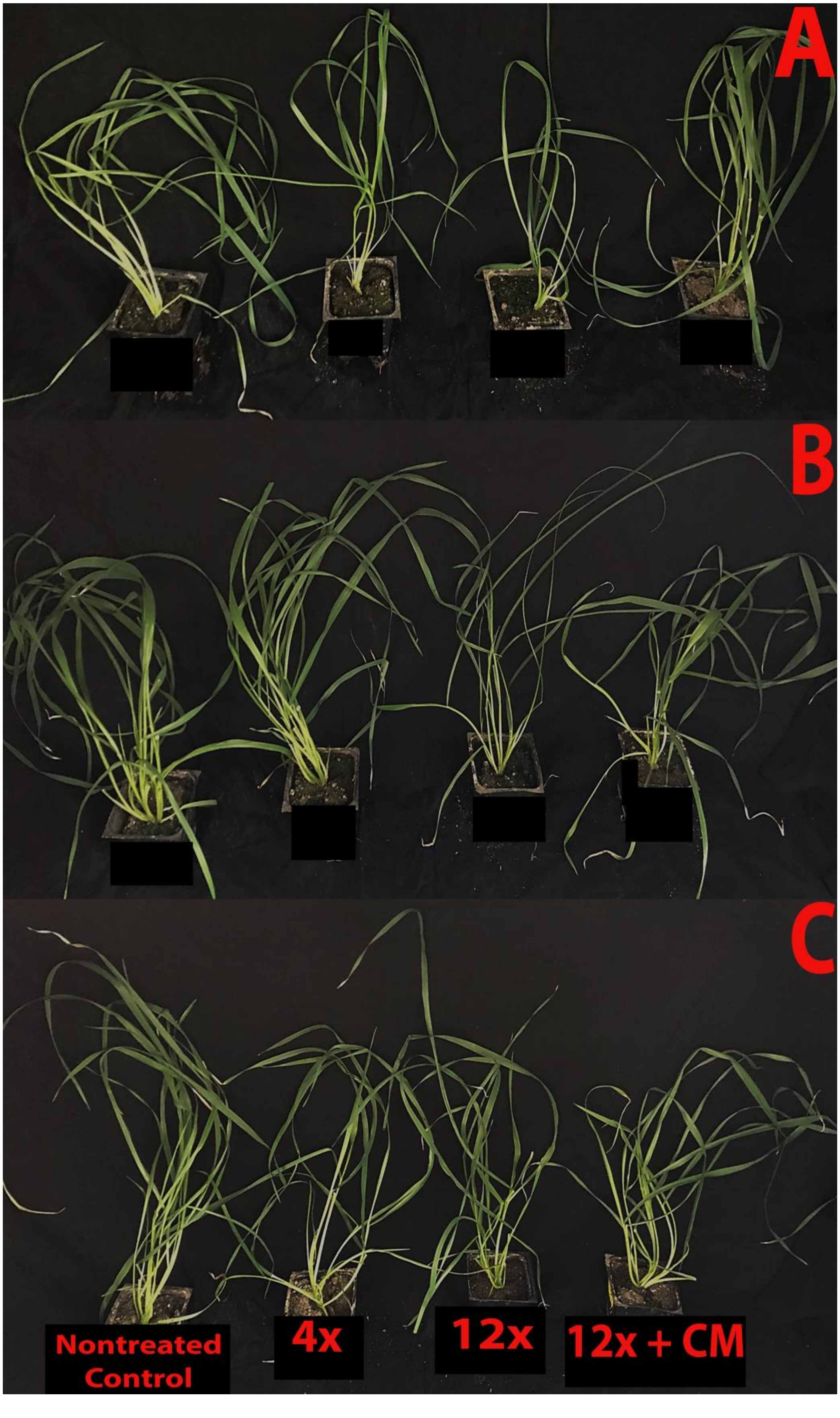
A
B
C
Nontreated Control
4x
12x
12x + CM

Supplementary Figure S2. Wheat group 5 alien substitution lines 20 days following herbicide or herbicide plus safener treatments. Treatments were applied to seedlings at Zadoks stages 11-12 included the nontreated control (adjuvants included in all other treatments: 1.25% methylated seed oil and 2.5% ammonium sulfate); 20 g a.e. ha^{-1} halauxifen-methyl (HM) (4x); 60 g a.e. ha^{-1} HM (12x); and 60 g a.e. ha^{-1} HM with 3.75 g ai ha^{-1} (equivalent to 60 µM; Quelex field rate) of cloquintocet-mexyl (CM) (12x + CM). Plants from the 5A, 5B and 5D alien substitution lines are displayed in panels **A**, **B**, and **C**, respectively.
